# Regulation of Estrogen Receptor ***α*** Expression in the Hypothalamus by Sex Steroids: Implication in the Regulation of Energy Homeostasis

**DOI:** 10.1155/2015/949085

**Published:** 2015-09-27

**Authors:** Xian Liu, Haifei Shi

**Affiliations:** Department of Biology, Miami University, 700 E. High Street, Oxford, OH 45056, USA

## Abstract

Sex differences exist in the complex regulation of energy homeostasis that utilizes central and peripheral systems. It is widely accepted that sex steroids, especially estrogens, are important physiological and pathological components in this sex-specific regulation. Estrogens exert their biological functions via estrogen receptors (ERs). ER*α*, a classic nuclear receptor, contributes to metabolic regulation and sexual behavior more than other ER subtypes. Physiological and molecular studies have identified multiple ER*α*-rich nuclei in the hypothalamus of the central nervous system (CNS) as sites of actions that mediate effects of estrogens. Much of our understanding of ER*α* regulation has been obtained using transgenic models such as ER*α* global or nuclei-specific knockout mice. A fundamental question concerning how ER*α* is regulated in wild-type animals, including humans, in response to alterations in steroid hormone levels, due to experimental manipulation (i.e., castration and hormone replacement) or physiological stages (i.e., puberty, pregnancy, and menopause), lacks consistent answers. This review discusses how different sex hormones affect ER*α* expression in the hypothalamus. This information will contribute to the knowledge of estrogen action in the CNS, further our understanding of discrepancies in correlation of altered sex hormone levels with metabolic disturbances when comparing both sexes, and improve health issues in postmenopausal women.

## 1. Introduction

Sex differences exist in the epidemic of obesity-related metabolic diseases. According to the recent National Center for Health Statistics [1], although there is no significant difference in obesity prevalence between men and women at any age, the incidence of obesity increases dramatically after women reach menopause. Additionally, the percentage of people who suffer metabolic diseases is particularly higher among men and postmenopausal women than in premenopausal women [1]. Further, obese premenopausal women have much less metabolic complications than obese men and postmenopausal women [1]. Among women, menopause is associated with a significant shift to an atherogenic lipid profile with elevated circulating level of triglyceride but lowered circulating level of high-density lipoprotein cholesterol [2, 3]. Consequently postmenopausal women are at an increased risk of developing visceral obesity, metabolic syndrome, and cardiovascular diseases independent of age, due to loss of endogenous ovarian hormone production [4]. Various types of hormone replacement therapy aiming to increase estrogen levels in postmenopausal women have been shown to ameliorate visceral obesity and lower risks for metabolic disorders and cardiovascular diseases [5]. Interestingly, elevated levels of circulating estrogens are associated with obesity and metabolic dysfunctions in men [6–9]. Increased estrogen levels are also seen in male rabbits with increased body fat and metabolic syndrome due to high-fat diet [10]. The causal relationship between sex steroid and obesity in males and females has not been determined yet [11, 12]. Therefore, insights into how altered sex steroid levels affect metabolic regulation are vital.

It is well-established that obesity is frequently associated with irregular menstrual cycles, decreased fertility, and altered patterns of various reproductive and metabolic hormones in humans [13] as well as in rodents [14]. Sex steroids play key roles in energy homeostasis and glucose regulation. Among sex steroids, including estrogens, androgens, and progestogens, deficit in levels of estrogens has been identified to be one of the major contributors to the metabolic syndrome and visceral lipid accumulation observed in postmenopausal women. In intact, young adult rodents [15] and young women [16] with regular estrous cycles, variation of feeding behavior follows natural fluctuation of estrogens such that, following the proestrus estradiol surge, daily food intake reaches its minimum during estrus, which is usually around 20% less than their maximum daily caloric intake during diestrus [17, 18]. In rodent models, ovariectomy (OVX), that is, removal of ovaries and thus majority of endogenous estrogens, is a typical experimental model for investigating metabolic disturbance due to estrogen deficiency. Increases in food intake, body weight, and body fat accumulation are usually observed following OVX, whereas exogenous, cyclic treatment of estradiol at physiological doses normalizes these changed variables to individuals with intact ovaries and normal estrous cycles [19–21]. Similar as in females, working estrogen signaling with normal range estrogen levels and functional estrogen receptors (ERs) is required for metabolic regulation in males. Male mice with deficient estrogen signaling due to either absence of aromatase, a key enzyme for converting androgen to estrogen, thus disrupting estrogen biosynthesis, or lack of receptors for estrogens develop similar phenotypes of increased adiposity, inflammation, and insulin resistance as individuals lack of estrogens [22–24]. Treatment of estrogen ameliorates hyperglycemia and insulin resistance in obese male mice [25]. In humans, men with congenital aromatase deficiency are insulin resistant, and estrogen replacement therapy has been successfully used to improve insulin sensitivity in these men [26]. Therefore, estrogens play critical roles in metabolic regulation in male and female animal models and humans. Understanding the metabolic regulation of estrogen's effects is of great interest to develop efficient therapy targeting on estrogen signaling to treat obesity and related metabolic disorders.

## 2. Estrogens Regulate Energy Balance in the Central Nervous System (CNS)

### 2.1. Hypothalamic Regulation of Energy Balance

The hypothalamus of the CNS is the integration center that controls homeostatic regulation of energy balance including energy intake and energy expenditure [27]. The major nuclei that contain neurotransmitter systems and circuits among neuronal and nonneuronal cells in the hypothalamus include the rostral part of medial preoptic hypothalamic area (MPOA) within the neural circuit for regulating thermogenesis [28] and the caudal medial basal hypothalamic region including the paraventricular nucleus of the hypothalamus (PVH) related to energy expenditure and HPA axis regulation, the arcuate nucleus (ARC), and the ventromedial nucleus of the hypothalamus (VMH) for controlling food intake (Figure 2). These nuclei are key brain areas in the neuroendocrine regulations of reproductive function and energy homeostasis. For instance, VMH-specific ablation of ERs abolishes sexual behavior in females [29] and males [30] in genetic mouse models, indicating that VMH is a critical brain nucleus that regulates reproductive function. The VMH is also recognized as a “satiety center” that suppresses appetite when VMH cells are stimulated [31] and induces hyperphagia when VMH cells are damaged [32]. Sex differences exist in the central regulation of energy balance and body weight. Sex steroid hormones influence the regulation of body weight, body fat, energy balance, and metabolism [33], which is at least partially achieved via ERs expressed in key neuropeptide-expressing neurons in the ARC and VMH of the hypothalamus [34–39].

### 2.2. Estrogens Regulate Energy Balance in the Hypothalamus

#### 2.2.1. Mechanisms of Estrogenic Action via ERs

Estrogenic activity is mediated through genomic along with nongenomic mechanisms. Like other sex steroids, estrogens exert their biological functions through binding to ERs, including classic nuclear ER*α* and ER*β*, membrane-localized nuclear ERs, and membrane-bound G protein-coupled ER (GPER, also known as GPR30) [40].

**Figure 1:**
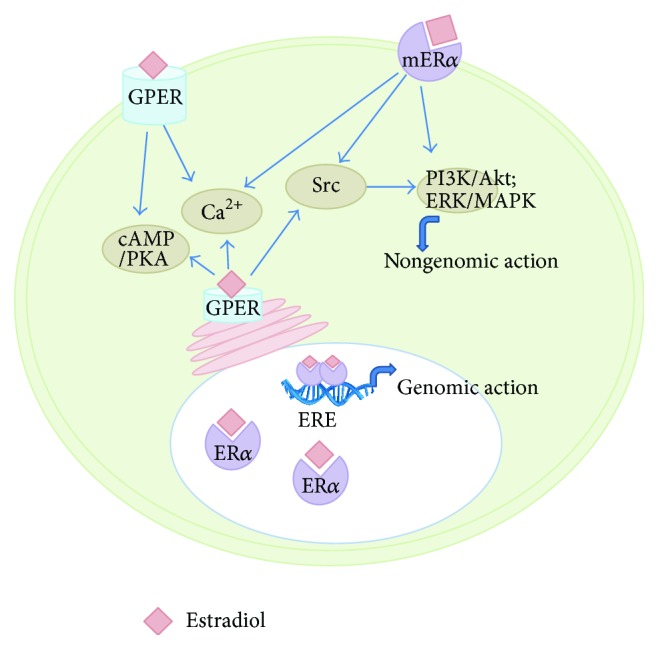
Schematic overview of estradiol-mediated genomic signaling pathway via nuclear ER*α* and rapid nongenomic signaling pathways via GPER and membrane isoform of ER*α*.

The classic nuclear receptors for sex steroid hormones, including estrogens, progestogens, and androgens, are activated by their respective steroid hormones and undergo conformational changes, dimerize with other hormone receptors, and recruit coactivator/corepressor molecules. The dimers then bind to their respective hormone response elements in the promoter of target genes and function as nuclear transcription factors, to alter transcription and expression of specific target hormone responsive genes and to mediate biological actions of their respective hormones [41, 42]. Such genomic activity between steroid hormones and their respective receptors typically occurs over a course of several hours to a day for the effects to be manifested, due to the time needed for transcription and translation of hormone responsive genes [43] (Figure 1). While the most abundant and potent female sex hormone for nuclear ER*α* is estradiol, ER*α* also binds to other natural forms of estrogens such as estrone and estriol with lower affinity than estradiol [44]. Additionally, many pharmacological, environmental, and food compounds are capable of binding ER*α*, either promoting or disturbing ER*α* genomic activity [45].

The importance of membrane steroid receptors is increasingly apparent. Besides nuclear-initiated genomic signaling via ER*α* and ER*β* localized inside nuclei, membrane-initiated nongenomic estrogenic signaling via membrane-bound ERs [46, 47] is also involved in the hypothalamic control of energy homeostasis [48, 49]. These membrane-localized ERs could be GPER or membrane isoforms of nuclear ERs. In order to differentiate which genes are translated to membrane-localized ERs, one could determine membrane ER expression in nuclear ER ablated genetic mouse model, or* vice versa*. Razandi et al. showed that no membrane ER could be detected in ER*α* and ER*β* double knockout mice [50], suggesting that membrane ERs and nuclear ERs are derived from the same genes. Kang et al. assessed transcription activity of nuclear ER*α* following knockdown or overexpression of membrane ER in breast cancer cells and reported a positive correlation in expressions between nuclear and membrane ERs. Specifically, lowered expression of nuclear ER*α* was detected when membrane ER expression was decreased and enhanced nuclear ER*α* expression was detected when membrane ER was increased [51]. Thus, the membrane-localized ER, at least in breast cancer cells, is a related isoform of the classical nuclear ER*α* [51]. Similar findings have been reported in the hypothalamic neurons and astrocytes obtained from male and female rats, as both a full-length isoform and a truncated variant of the ER*α* have been detected using surface biotinylation and immunocytochemistry [52, 53]. Subsequent studies have shown that about 3–5% of classical nuclear ER*α* is distributed on membrane [54, 55]. These studies suggest that membrane-localized ER could be a variant of classical nuclear ER. A currently accepted view is that GPER and membrane-localized ER*α* are both presented in cell membrane. One possibility is that collaboration between membrane-localized ER*α* and GPER exists, and GPER could induce the expression of the membrane-localized ER*α* [51]. These studies have collectively demonstrated that a subpopulation of nuclear ER*α* is present at the plasma membrane in many cell types within the CNS, including the hypothalamus.

Nongenomic estrogenic signaling occurs more rapidly than the classical genomic estrogenic signaling, typically over a short course of seconds to minutes [56]. This nongenomic signaling starts at membrane, activates several kinase cascade pathways and triggers intracellular signaling, and subsequently induces multiple actions such as gene transcription and alteration in activity of neurons in the CNS. Extranuclear and membrane-associated isoforms of ERs can congregate with signaling molecules, such as G proteins and nonreceptor tyrosine kinases (Src) to facilitate interaction and rapid intracellular signaling [49]. Binding of estrogens and nonnuclear ERs may induce several intracellular signaling kinase cascade pathways, including stimulation of adenylyl cyclase activity and cAMP-dependent protein kinase (PKA) pathway and cAMP-response element binding protein signaling cascade [57, 58], mobilization of intracellular Ca^2+^-dependent protein kinase C (PKC) pathway [59–61], activation of extracellular signal-regulated kinase (ERK)/mitogen-activated protein kinase (MAPK) pathway [62–65], and activation of receptor tyrosine kinase and phosphatidylinositol 3-kinase (PI3K)/Akt signaling pathway [66–68] (Figure 1). These abovementioned intracellular signaling events induce rapid actions that mediate a variety of estrogenic actions besides regulation of energy homeostasis and body fat, such as neuronal excitability, neuronal protection, reductions in inflammation, and neurite outgrowth [69].

In order to distinguish involvement of classical versus nonclassical ER*α* signaling in metabolic regulation of energy balance, a recent study investigated* in vivo* physiology using an ER*α* knockout mouse model with manipulation in nonclassical and classical estrogen signaling. Briefly, a mutant allele with disrupted estrogen response element (ERE) is introduced into the nuclear ER*α* knockout mouse model. Consequently, these ER*α* knockout mice express a mutant ER*α* with intact nonclassical signaling capacity but disrupted classical estrogen signaling capacity; thus ER*α* only signals via nongenomic pathway in this mouse model [70]. Interestingly, all of the metabolic dysfunctions presented in ER*α* knockout mice are normalized in these mice with nonclassical signaling but lack of genomic estrogen signaling [70], confirming that nonclassical estrogen signaling is a significant player that mediates the metabolic effects of estrogens. Therefore, estradiol and other ER*α* ligands at least act via membrane-localized ER*α* to regulate energy homeostasis.

#### 2.2.2. Estrogenic Action on Energy Balance in the Hypothalamus via ERs

The hypothalamus is structurally and functionally sexually dimorphic [71] and is classically recognized as a steroid hormone-responsive area of the brain [72]. It is important that the brain remains sensitive to steroid hormones during postnatal development period even during adulthood, which is critical for several estrogen-dependent behaviors [73]. When acting in the hypothalamus, estrogens decrease food intake and increase energy expenditure [74], suppress lipogenesis [75, 76], and improve glucose metabolism [77]. Among all the ERs, ER*α* is considered to be the receptor primarily responsible for energy homeostatic modulation in the CNS and in the periphery [40, 49]. ER*α*-containing neuronal and nonneuronal cells are mostly located and much more abundant than other types of ERs in the ARC and the ventrolateral part of the VMH [78–81], the key brain nuclei regulating energy homeostasis. Recent studies have indicated that regulation of ER*α* expression in these nuclei by steroid hormones could provide direct mechanisms underlying how estrogens affect the activity of these brain nuclei to regulate energy balance, body fat distribution, and metabolism [34–39, 82].

Deletion of ER*α*, either whole-body knockout [23, 83] or hypothalamic nuclei-specific knockout [37, 84], increases adiposity and causes the metabolic syndrome in male and female mice. It is noteworthy that suppression of ER*α* gene expression in the steroidogenic factor-1 (SF-1) neurons in the VMH or in the proopiomelanocortin (POMC) neurons in the ARC has a greater impact in females than males to increase food intake and body fat and to decrease energy expenditure and metabolism [37, 84]. These findings not only expand our understanding of the metabolic regulation of ER*α* in the hypothalamus but also support that ER*α* in the neurons in the VMH and ARC, at least SF-1 and POMC expressing neurons, plays important roles in regulating energy balance, especially in females.

#### 2.2.3. ER*α* in Nonneuronal Cells in the Hypothalamus

There are more nonneuronal cells than neuronal cells within the CNS. Nonneuronal cells encompass diverse cell types, with microglia and astrocytes being the most abundant cell types. Regulation of energy homeostasis involving neuronal cells has been focused in majority studies previously, while microglia and astrocytes have been less studied. Two key points are pertinent to the topic reviewed here. First, microglia and astrocytes locate in the hypothalamic regions that respond to steroid hormones including estrogens [85–87]. Microglia and astrocytes are well-documented for their contributions to estradiol-induced effects in neurogenesis and synaptogenesis, synaptic plasticity, and neural repair [88, 89] sexual differentiation of the brain [90, 91], modulation of estrogen-positive feedback in LH surge [92], and maintaining function of neural circuits among neighboring nonneuronal and neuronal cells during developmental stage and adulthood [91, 93]. Second, the significance of hypothalamic microglia and astrocytes in pathological metabolic processes associated with obesity, such as hypothalamic inflammation [94–98] and related insulin and leptin resistance [99–101], has become increasingly evident in rodent and human disease models. The ARC and the VMH of the hypothalamus are sexually dimorphic brain nuclei that control reproduction [102] and energy homeostasis [103], which contain ERs with ER*α* at a much higher concentration than ER*β* [104]. In fact, hypothalamic inflammation with activation of microglia and astrocytes in these brain nuclei caused by high-fat diet feeding and increased body fat have been demonstrated in recent studies [97, 100, 105]. Therefore, it is logical to presume the presence of ERs in hypothalamic microglia and astrocytes to contribute to anti-inflammatory effects of estrogens and differential regulation of energy homeostasis between sexes [33, 106].

Whether ER*α* is expressed in microglia and astrocytes in the hypothalamus has been an ongoing debate. An early study observed ER*α* immunoreactivity in neuronal cells only but not in any nonneuronal glial cells including microglia and astrocytes [107]. Regarding ER expression in microglia, the above notion was questioned as ER*α* presence was demonstrated in primary microglial cell culture established from the forebrains of neonatal rats [108] and ER*α* was identified as the receptor modulating microglial activity [109]. A few recent studies, however, confirmed the absence of ER*α* protein and gene expression in primary mouse and human microglia [110–112], whereas ER*β* expression was abundant in microglia [112]. Regarding ER expression in astrocytes, some* in vitro* studies have reported that both ER*α* and ER*β* are expressed intracellularly and on plasma membrane of astrocytes from cultured CNS cells [86, 89, 113–116], although one study reported that ER*β* expression in astrocytes was uncertain [117]. Recently, ER*α* has been found in primary hypothalamic astrocytes cultured from male and female pups [53, 110, 118]. Using ER*α* knockout and ER*β* knockout mice, Mong and Blutstein have demonstrated that ER*α* but not ER*β* mediates the action of estradiol on astrocytes in the ARC [87], suggesting that, at least in the ARC, ER*α* is the receptor mediating the actions of estradiol in astrocyte morphology. To reconcile the literature, most studies on ER expression in microglia and astrocytes have been conducted using* in vitro* cultures and have demonstrated dominant ER*α* expression in astrocytes and dominant ER*β* expression in microglia. Future* in vivo* demonstration of ERs in hypothalamic microglia and astrocytes in neonatal and adult brains and assessment of their physiological function in metabolic regulation are needed to further our knowledge of estrogenic metabolic regulation in the CNS.

#### 2.2.4. Regulation of ER*α* Expression in the Hypothalamus

**Figure 2:**
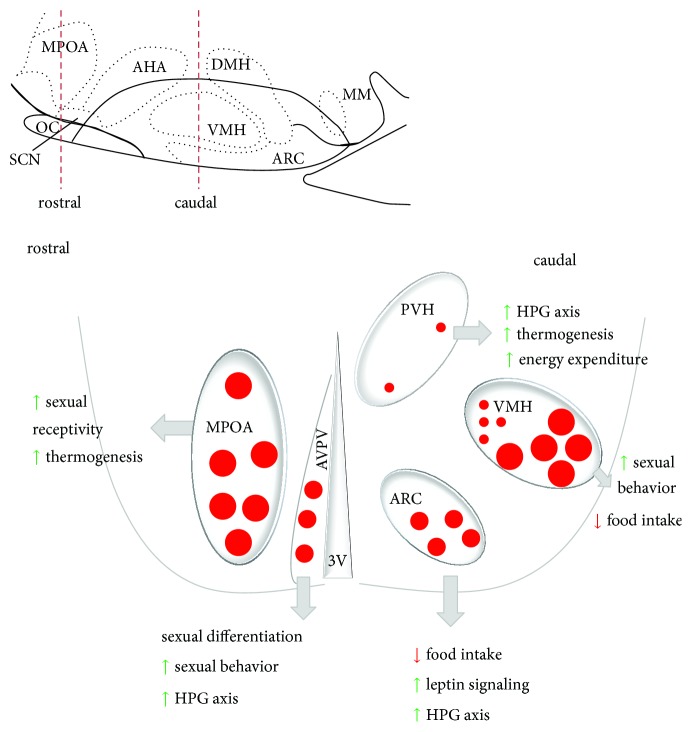
Distribution of ER*α* in the hypothalamus and their physiological effects. Schematic coronal section showing locations of major nuclei in the rostral (left) and caudal (right) hypothalamus containing ER*α*-expressing cells, the relative quantities of ER*α* in these nuclei (large red dots indicate abundant distribution and small red dots indicate scarce distribution), and their physiology effects in reproduction and energy homeostasis.

ER*α* distribution in the hypothalamus is highly conversed across a number of species, including rat [81, 104, 119], mouse [79], hamster [120], guinea pig [121], ferret [122], opossum [123], musk shrew [124], bird [125, 126], cow [127], nonhuman primates at pubertal stage [128] and adulthood [129, 130], and adult human [131–133]. ER*α* is abundantly distributed in several key brain regions implicated in regulating energy homeostasis in both males and females, including the ventral lateral portion of the VMH and the ARC related to the control of food intake, the PVH related to energy expenditure and HPA axis regulation, the MPOA that influences thermogenesis, and the anteroventral periventricular nucleus (AVPV) regulating sexual behavior and HPG axis [78, 81, 104, 134] (Figure 2). In general, males express less amount of ER*α* than females in these key hypothalamic nuclei involved in metabolic regulation. The gene expression, protein levels, and distribution pattern of ER*α* in the hypothalamus can be modified by alterations in circulating steroid hormone levels. Hormone levels can be experimentally manipulated using methods of surgical removal of gonads and hormone replacement, or fluctuate due to natural variation across estrous cycles and during certain physiological stages such as puberty, menopause and pregnancy. The degrees to which the reported differences, either between species or within one species, are indeed due to differences in sex or hormone status remains vague from the current literature.

The exact mechanism regulating ER*α* expression in the hypothalamus is largely uncertain but at least in part could be at the mRNA level, as seen in breast cancer cell lines with elevated ER*α* expression [135]. Recent studies have indicated that the genomic organization pattern of ER*α* gene (Esr1) is more complex than it was previously presumed, especially in humans [136]. Specifically, Esr1 gene possesses promoters in its 5′ end untranslated region, which could generate several mRNA splice variants to encode the same protein ER*α* [137, 138]. This multiple promoter system is believed to play a role in the tissue-specific and temporal regulation of ER*α* gene expression. For example, both human ER*α* promoters A and C are used in human breast adenocarcinoma cells MCF7 but only promoter A is used in human breast carcinoma cells ZR-75-1 [139]. Considering hypothalamus is a heterogeneous region in which nucleus groups have different origins and functions, it is highly possible that various nuclei utilize distinct promoters or promoter combinations of Esr1 among one another at different stages of development. Usage of multiple promoters of Esr1 changes an array of transcription activators and repressors and leads to variation of alternative splicing of mRNA transcripts while encoding the same full-length protein ER*α*. Furthermore, Esr1 promoter region has been shown to undergo epigenetic modification, that is, DNA methylation, under normal and pathological conditions [140]. It is possible that, by changing the length and/or sequence of 5′ end untranslated region, the stability of a specific transcript can be enhanced or compromised. The regulation of ER protein is also complicated, as ER mRNA is not always translated into functional ER protein, possibly due to brain region-specific posttranscriptional processing [141].

In this review, we gather evidence with regard to how expression and distribution of ER*α* in the hypothalamus of the CNS are regulated by various sex hormones in both males and females from human and animal studies and discuss discrepancy in current literature. This information will contribute to understanding of estrogenic action in the CNS and improve health issues in postmenopausal women.

## 3. Regulation of ER*α* Expression by Sex Steroids

Sex steroid hormones profoundly affect metabolic regulation of many species. Although the distribution pattern of ER-containing cells in the hypothalamus is similar across species, the regulation of ER expression could be relatively dynamic depending on circulating sex hormone levels and reproductive status, indicating the plasticity in ER*α* regulation. In men, androgen levels rise during puberty and decrease gradually over the lifespan [142]. In contrast, from puberty through menopause, women continuously experience cycles of fluctuations of estrogens and progestogens in their lives, and all these sex hormones decrease suddenly following menopause [143, 144]. Additionally, the levels of progestogens rise rapidly following mating, and both estrogens and progestogens are at sustained high levels during pregnancy. Towards the end of pregnancy, progestogen levels sharply decline whereas estrogen levels increase, which allows parturition and maternal behavior to be displayed [143, 144].

All circulating androgens in men and most of the estrogens and progestogens in premenopausal women are produced in the gonads, testes in males and ovaries in females. On the other hand, circulating estrogens and progestogens in men, androgens in premenopausal women, and all sex steroids in postmenopausal women are synthesized in tissues other than gonads, including the brain, adipose tissues, and adrenal glands. The sex steroids secreted by nongonad tissues may also act locally as intracrine or paracrine factors, distinct from endocrine hormones. Testosterone and 5*α*-dihydrotestosterone, estradiol, and progesterone are the most potent respective androgens, estrogens, and progestogens that carry out physiological functions.

Regulation of ER*α* expression in the hypothalamus due to dynamic endocrine status and changes in circulating steroid levels could be region-specific and different among species, depending on dissimilar patterns of reproductive cycles (see below). It is noteworthy, however, that ER*α* expression could be regulated by organizational effects during developing stages rather than activational effects influenced by steroid hormones. This is demonstrated by a study using castrated male and female rats, both of which lack majority of endogenous sex hormones [145]. Lauber et al. reported that the castrated male rats had significantly lower amount of ER*α* mRNA levels, less than half, than OVX females in the VMH and the ARC [145]. Such finding suggests a sex difference in basal, constitutive ER*α* expression level in the rat hypothalamus since there is no impact of circulating sex hormones in either sex. Thus sex difference in ER*α* expression may not be merely due to sex hormones. Consequently, data on the sex steroid hormone regulation of sex steroid receptor expression must be interpreted with care.

Differential ER*α* expression would affect the sensitivity to steroid hormones and consequently affect their endocrine physiology and behavior, which contribute to the adaptation of neuroendocrine regulation of energy homeostasis in obesogenic environment. In this section, we focus on discussing steroidal regulation of hypothalamic ER*α* expression in the context of metabolic regulation of energy homeostasis.

### 3.1. Estradiol Regulates ER*α* Expression in the Hypothalamus

ER*α* expression in the hypothalamus changes with estrous cycle as a function of circulating levels of estradiol and progesterone [119, 145–149]. Various animal and human studies have demonstrated that ER*α* expression can be down- or upregulated by circulating levels of estrogens in a brain region-dependent manner across species [122, 150–152]. For example, estradiol increases ER*α* mRNA expression in the VMH, decreases ER*α* mRNA expression in the lateral septum, and has no effect on ER*α* mRNA expression in the dorsal hypothalamus in lizards [150]. In a separate study using male ferrets, ER*α* immunoreactivity is reduced in the MPOA, is increased in the medial VMH, and is unchanged in the lateral VMH and the ARC by estradiol treatment [122]. Therefore, regulation of ER*α* in the hypothalamus by estradiol, either at expression level or protein level, is regulated in a brain nuclei-specific manner.

It is generally assumed that estradiol would downregulate its own receptor ER*α*, similar to what many other hormones do. In agreement with this, several groups have shown that removal of majority of endogenous estradiol by OVX increases the number of ER*α* mRNA-positive neurons in the VMH of the hypothalamus of female rats [146, 147]. Accordingly, OVX female or castrated male rats receiving either an acute [146, 153] or chronic [154] treatment of estradiol benzoate up to 1 mg [153] have decreased levels of ER*α* mRNA in the hypothalamus. Specifically, ER*α* mRNA levels decline in the VMH and ARC 18–24 hours after estradiol benzoate treatment [153, 154], and the numbers of ER*α* positive neurons reduce in the VMH and anterior hypothalamic area following a 2-week treatment of estradiol benzoate at pharmacologic dosages [154]. Thus, it seems that, at least in rats that were treated with pharmacologic level of estradiol, hypothalamic ER*α* mRNA and protein levels are inversely correlated with circulating estradiol concentration to maintain responsiveness to hormonal activation in ER-containing cells, which would ensure a balanced, homeostatic system to prevent excessive estrogenic action [145–147] (Table 1).

On the contrary, other groups have shown opposite results (Table 1). When either physiological or supraphysiological concentrations of estradiol are utilized to treat primary neurons isolated from the ventrolateral area of the VMH of young female rats, measurement of ER*α* using immunofluorescence reveals that estradiol increases the protein level of ER*α* in these primary neurons* in vitro* [155], suggesting a positive relationship between estradiol level and ER*α* expression. Findings from several* in vivo* studies have reached this same conclusion. When compared with ovarian intact, cycling females, males with lower endogenous estrogen levels than females express much less amount of ER*α* in the VMH [156, 157] or in the MPOA [152] seen in females. A recent study confirmed the importance of the dosage of estradiol treatment on ER*α* expression in the ARC [158]. OVX rats are injected with two different doses of estradiol, either at a pharmacologic dose of 50 *μ*g or at a physiological dose of 2 *μ*g. Not surprisingly, ER*α* expression in the ARC is significantly downregulated in the rats treated with the pharmacologic dose of estradiol but is not changed in the rats treated with the physiological dose of estradiol [158]. Devidze et al. [157] have demonstrated that the mRNA level of ER*α* within the VMH is increased by a two-day exposure to a physiological dose of estradiol that is much lower than the dosage of estradiol used in earlier studies. Consequently, ER*α* expression is greater in OVX rats with estradiol treatment than their control OVX rats receiving vehicle treatment. Indeed, the quantity of ER*α* in the VMH gradually rises in response to increasing concentrations of estradiol throughout the estrous cycle until it reaches its maximum during the proestrus [148]. Similarly, a human study that compared ER*α* immunoreactivity in young adult men and women has shown stronger ER*α* immunoreactivity in the VMH of female brains than male brains [131]. The physiological relevance of this ER*α* elevation by increased estradiol level is to reinforce and augment estrogenic action during active phase of reproductive cycle for regulating related metabolism and reproductive behavior and ultimately leads to the survival of individuals and species. This notion of promoting behavioral regulation by estrogenic action is indeed supported by an abovementioned chronic study when 5-day parenting experiment is paired with treatment of estradiol benzoate; there is an increase in the number of ER*α* positive cells in the VMH [154]. Such increased ER*α* expression provides a means for estrogens to carry out their physiological and behavioral effects critical for reproductive function and success.

The regulation of ER*α* mRNA by estradiol also could differ among species. For example, estradiol treatment decreases ER*α* mRNA in the VMH in female rats [146, 147] but increases ER*α* mRNA in the VMH in female lizards [150, 159]. The species difference between rats and lizards could be due to their distinct reproductive physiology [160]. Briefly, the duration of follicular phase is brief in rats; thus estradiol surge during proestrus is immediately followed by behavioral estrus and onset of sexual receptivity with declining estradiol level. Therefore, in female rodents, increasing ER*α* expression in response to falling estradiol level is necessary for maintaining neural sensitivity after ovulation when individuals are sexually receptive. In contrast, the duration of follicular phase in lizards is much longer and extends to overlap with behavioral estrus. As a result, the onset of sexual receptivity occurs when estradiol level is still high in lizards, as well as many other vertebrates including rabbits, dogs, cats, bears, and primates. Therefore, lizards and many other mammal species need enhanced neural sensitivity during these latter stages of follicular phase when circulating estradiol level is high and individuals are sexually receptive, attributed to the increase in ER*α* expression in response to initial estradiol surge during early stages of follicular growth in these species.

Differences in the regulation of ER*α* expression have also been reported between the sexes, but such observed sex difference is not consistent among different studies using similar* in situ* hybridization histochemistry method. Lauber et al. reported that estradiol reduced ER*α* gene expression in the VMH and the ARC of OVX female laboratory rats, but estradiol failed to regulate ER*α* expression in castrated male rats [145]. In contrast, Simerly and Young reported comparable reduction of ER*α* gene expression in the VMH and the ARC between castrated male rats and OVX and estrous female rats [147].

To summarize, the abovementioned studies strongly suggest that the mRNA and protein levels of ER*α* within the key brain nuclei are positively correlated with circulating estradiol levels within physiological ranges, whereas they are negatively correlated with estradiol levels if estradiol levels reach pharmacologic levels. The reason for such discrepancy is not clear when considering the massive degree of complexity of living animals. We speculate that several factors may be involved. First, the response of neurons in these hypothalamic nuclei might be multiphasic, which is regulated by a negative feedback mechanism. Since there is no feedback control leading to downregulation when estradiol directly acts on isolated neurons* in vitro*, it is likely that ER*α* in these isolated VMH neurons neither autoregulate its own expression nor are controlled by any feedback mechanism, as seen in some other cell lines including cancer cells [161], osteoblasts [162], and macrophages [163]. The second possibility for such discrepancy involves the dosages and methods adopted to introduce estradiol among different studies. Using supraphysiological or pharmacologic dose of any hormone is likely to induce adverse impact or hyperstimulation to neurons, whose effect is of low physiological significance [164]. Besides using different concentrations of estradiol, different methods have been used to replace estradiol. Chronic, prolonged estradiol capsule treatment potentially desensitizes neurons, which may disable feedback mechanisms and result in uncontrolled adaptation. Third, the effects of estradiol might be concealed by another major ovarian hormone, progesterone, which is also being removed by OVX. When considering progesterone, it is not surprising that we observe different results when adult mature females are compared with young females that do not cycle regularly [157] or* in vitro* isolated neurons [155]. Furthermore, it is worth noticing that a number of factors that may be implicated, including aging [165, 166] and chronic exposure to environmental factors such as bisphenol-A [167, 168], are capable of altering endogenous ER*α* and E2 level as well. Thus the discrepancies among different studies may reflect the differences in ages, composition of diet, or dietary origins.

### 3.2. Progesterone Regulates ER*α* Expression in the Hypothalamus

Progesterone is another sex steroid that is mainly synthesized and secreted in the ovaries and the adrenal gland. During vertebrate ovarian cycles in primates and rodents, growing follicles secrete increasing level of estrogens during latter stage of diestrus which peaks at proestrus and declines following ovulation, while progesterone level increases following the luteinizing hormone (LH) surge, ovulation, and formation of the corpus luteum during diestrus and proestrus [169]. The elevation in plasma progesterone during diestrus is eliminated following adrenalectomy, confirming the adrenal origin of progesterone [169].

Progesterone is an important modulator of estrogenic action in the CNS and it affects LH release and ovulation in a biphasic manner [170]. The role of progesterone on estrogenic function is complicated, as it can be either inhibitory or stimulatory depending on the timing and sequence of injections [171]. For example, in OVX rats, estradiol injection alone can induce daily surges of LH [172]. These daily LH surges are blocked by progesterone treatment [173]. Thus, one of the functions of the increased progesterone secretion in the proestrus is to abolish LH surge [173]. On the contrary, treatment with progesterone following estrogen priming, which mimics the estrogen peak that precedes progesterone elevation in cycling females, activates sexual behavior [171]. Therefore, sequential secretion of estrogens and progestogens during ovarian cycles determines timing of ovulation and leads to the onset of a series of sexual behavior [171].

The theory of progesterone regulation on ER*α* expression is supported by the fact that both progesterone receptor (PR) and ER*α* are coexpressed in certain nuclei in the CNS [174]. Blaustein and Brown [175] have reported that when estradiol-treated OVX female rats are injected with progesterone* in vivo*, ER*α* binding is significantly reduced in the anterior pituitary, the MPOA, and the medial basal hypothalamus where the VMH and the ARC are located. Such progesterone's effect on ER*α* expression is not evenly displayed among the brain areas with coexpression of PR and ER*α*. Additionally, when progesterone and estradiol are treated together to primary disassociated VMH cells* in vitro*, physiological concentrations of progesterone counteract estradiol-induced elevation of ER*α* expression, whereas progesterone treatment alone upregulates ER*α* expression [155] (Table 1). One possible explanation for the dual effects of progesterone is interinhibition of transcriptional activities, due to activated PRs localizing to the progesterone response element (PRE) in the promoter region of ERE. Thus, simultaneous activation of both receptors may lead to protein-protein interaction that eventually blocks the activation site for estrogens, causing counteraction between progesterone and estradiol. Furthermore, under certain conditions when PR ligands do not activate transcription, PR isoforms potently suppress transcriptional activity of ER in cultured primary uterine cells from rats [176]. It is also possible that binding of PR at PRE may not be required for upregulation of ER*α* expression by progesterone. PR agonist can increase expression of reporter gene in the cells artificially expressing PR or ERs and transfected with vectors containing ERE but not PRE in their promoters [177]. It is mentioned earlier in this review that ER*α* expression increases after OVX in many species, which could be at least partially due to the removal of progesterone by OVX and thus release inhibition of progesterone on ER*α* expression [176]. Alternatively, decrease in progesterone level following OVX might also attenuate progesterone inhibition on other factors. For example, decrease in progesterone concentration increases the amount of neurotransmitter norepinephrine in the CNS, which has been shown to augment number of ERs in the hypothalamus [178] as well as lordosis behavior [179] in guinea pigs. The aforementioned studies collectively indicate that when treated alone, progesterone upregulates ER*α* expression, while when cotreated with estrogens, progesterone downregulates ER*α* expression.

Progesterone has these similar effects on the regulation of ER*α* expression during gestation. In rodents, progesterone level rapidly increases following mating and reaches plateaus during gestation days 15–20 and then it sharply declines, whereas estradiol level increases towards the end of pregnancy after day 20, a shift allowing parturition and maternal behavior to be displayed. Levels of progesterone and estradiol change naturally but dramatically during gestation and thus can be used as a naturally occurring model to study impact of progesterone and estradiol on the expression of ER*α*. During gestation, ER*α* expression in the ARC remains constant, except for an increase in ER*α* expression on days 12 and 19 of gestation when progesterone level reaches its plateaus before rise of estrogens level. Consequently, ER*α* expression in the ARC on days 12 and 19 of gestation is greater compared with ER*α* expression of diestrus rats and rats at postpartum lactation when their estrogens levels are climbing but progesterone levels are declining [180]. Similarly, Mann and Babb [181] have reported that ER*α* mRNA levels are constant between days 15 and 21 of gestation when progesterone level reaches the peak.

To summarize, ER*α* expression increases when progesterone level is high and estrogen level is low, as seen in animals treated with progesterone alone or during gestation; ER*α* expression decreases in the presence of progesterone when animals are cotreated with estrogens or during postpartum phase when estrogen level sharply rises.

### 3.3. Androgens Regulate ER*α* Expression in the Hypothalamus

Androgens influence gene transcription through activation of androgen receptor (AR), a type of nuclear receptor that shares similar genomic signaling mechanism as ER and PR. Briefly, upon ligand binding, AR dimerizes and binds with specific DNA motifs androgen response element (ARE) in its target genes [41]. In males, testosterone's function mediated by AR has antiobesity features. Testosterone deprivation in men contributes to the development of metabolic syndrome. Male mice lacking AR develop insulin resistance, leptin resistance, and late onset obesity primarily due to decrease in locomotor activity, a component of energy expenditure [182, 183]. Although male sexual behavior is mediated by androgens, male reproductive behavior is also influenced by estradiol transformed by testosterone aromatization in the brain.

Castration and thus reduced androgen levels have been reported to increase ER*α* mRNA in the MPOA [119, 151, 184] and in the VMH [147] of male rats and male mandarin voles [185], suggesting that ER*α* expression in the CNS is inhibited by androgens. Findings by Wu and Gore support this notion and demonstrate that, in the MPOA, ER*α* cell density is substantially higher in vehicle-treated compared with testosterone-treated castrated rats of both young and old ages [186]. Additionally, in the AVPV, testosterone decreases ER*α* cell density but to a lesser extent [186]. Furthermore, androgens also affect ER*α* expression in females. Testosterone treatment attenuates ER*α* mRNA expression in the VMH and ARC [147] and in the MPOA [151] in both males and females. Thus ER*α* expression in the hypothalamus is greater in females than in males [145], increased by castration in males, and inhibited by androgen treatment in both males and females, indicating that ER expression in the hypothalamus is downregulated by circulating androgens in both sexes (Table 1).

Similarly both men and women could suffer from various forms of metabolic dysfunction and abnormalities due to androgen imbalance, including hypoandrogenism in aging men [187, 188] and hyperandrogenism in women with polycystic ovary syndrome (PCOS) [187, 189]. Lowered circulating levels of androgens in men are associated with insulin resistance and obesity, while testosterone treatment in hypogonadal men improves insulin sensitivity and reduces body fat content [187, 188, 190]. The functional role of androgens in female energy metabolism is not well-characterized and has not been directly tested yet, but it is suggested from human studies with women suffering PCOS or animal studies using a PCOS animal model induced by injection of letrozole, a third-generation aromatase inhibitor. Androgen excess in women, one of the key diagnostic characteristics of PCOS [191], is associated with insulin resistance and obesity [187, 189]. Briefly, women with POCS [192–194] or female rats of PCOS model [195] have abnormally low levels of estrogens but high levels of androgens due to exaggerated androgen synthesis from the ovaries and adrenal glands. Additionally, PCOS women develop central visceral obesity, due to the fact that androgens promote abdominal fat deposition. In a letrozole-induced PCOS animal model, letrozole treatment decreases estradiol secretion and ER*α* expression in the ovaries of female rats [195] and decreases estradiol content and ER*α* expression in the hippocampus of male mice [196]. It is not clear whether or not ER*α* expression in the hypothalamus changes in females with high androgen levels.

There are two possible mechanisms through which androgen treatment may regulate ER*α* expression: through its aromatization to estradiol or through binding to ARs and inducing genomic changes in AR and ER*α* coexpressing neurons. The former possibility has been tested by DonCarlos et al., as aromatizable androgen testosterone but not nonaromatizable androgen dihydrotestosterone downregulates the levels of ER mRNA in the POA [151], confirming that estrogen derived from aromatization of testosterone suppresses ER expression in the hypothalamus.

## 4. Conclusion

Sex differences exist in the prevalence of obesity and related metabolic diseases [1]. Sex steroids, especially estrogens, contribute critically in the regulation of energy homeostasis and play protective roles in the development of metabolic complications. Menopause, characterized by a sudden reduction in female sex hormones, leads to visceral lipid deposition and increased risk in metabolic disorders [197]. Discrepancies exist however in the clinical correlation of altered sex hormone levels with metabolic disturbances when comparing the two sexes. Some studies have shown that hormone replacement therapy in postmenopausal women has positive effects on treating visceral obesity and related metabolic diseases [198–200], while other studies have failed to confirm the positive effects [201–203] and even questioned beneficial effects and safety of hormone replacement therapy [204]. Understanding estrogenic actions is required when developing safe and effective hormone replacement therapy.

Recent basic, translational, and clinic studies have greatly advanced our understanding of the mechanism underlying estrogenic effects. Estrogens utilize both genomic and nongenomic signaling mechanisms for estrogenic effects in the central and peripheral systems, primarily via ER*α* [40]. ER*α* is abundantly located in the MPOA, the VMH, and the ARC of the hypothalamus [78, 81, 104]. It is well-accepted that all major sex steroid hormones regulate ER*α* expression in the CNS. In this review, three understudied areas are identified that await further investigation.

First, inconsistency exists in the literature regarding the regulation of estrogen receptors by manipulation of sex hormones. The discrepancy exists in the literature due to different model systems, dosages of hormones, and experimental paradigms being used for testing. It is necessary to gain a more thorough understanding of estrogenic action in order to improve hormone therapeutics in humans. The consensus is that, within the physiological ranges, estrogens or progestogens alone increase ER*α* expression in the hypothalamus (Table 1), whereas androgens or progestogens in combination with estrogens inhibit ER*α* expression in the hypothalamus. Such effects would disappear or even become opposite if the paradigm and/or dosage are changed. Thus, dosages and combination of hormones are critical components for safe and effective hormone replacement therapies. Second, the importance of membrane steroid receptors is evident. The relationship between genomic and nongenomic estrogenic actions via respective nuclear and membrane-bound estrogen receptors, especially in* in vivo* physiology and biological functions, is uncertain, due to the fact that limited research has been done. The time courses of genomic and nongenomic estrogenic effects are dramatically different [56]. Understanding the coordination between nuclear and membrane estrogen receptors and their respective intracellular signaling pathways would contribute to developing more effective hormone therapies in future. Third, the actions of ERs in hypothalamic nonneuronal cells, such as microglia and astrocytes, are basically unknown, although it is clear that these cells play critical roles in regulating reproductive functions and metabolic processes and they are action targets for estrogens. Limited literature suggests that different ER subtypes play distinct roles in microglia and astrocytes, with ER*α* being dominant in astrocytes and ER*β* being dominant in microglia. Future* in vivo* studies are needed to assess their physiological function in regulating reproduction and metabolism.

## Figures and Tables

**Table 1:** Regulation of ER*α* in the hypothalamus by treatment of steroid hormones in male and female rats.

Hormones	ER*α* expression	Sex	References
Estradiol	Increase	Female	Cultured VMH cells: [155] VMH: [157]
		Male	VMH: [157]
	Decrease	Female	ARC: [145–147, 153, 158] (high dose) VMH: [78, 145–147, 153] AVPV: [153] MPOA: [148]
		Male	ARC: [147, 153] VMH: [153]
	No change	Female	VMH: [145] ARC: [145] AVPV: [153]
		Male	VMH: [158] (low dose)

Progesterone	Progesterone + estradiol decrease	Female	MPOA and medial basal hypothalamus: [175] Cultured VMH cells: [155]
	Progesterone alone increase	Female	Cultured VMH cells: [155]

Testosterone	Decrease	Female	VMH and ARC: [147]
		Male	MPOA: [184, 186] AVPV: [186] VMH and ARC: [147]

## Abbreviations

AR: Androgen receptor
ARE: Androgen response element
ARC: Arcuate nucleus
AVPV: Anteroventral periventricular nucleus
CNS: Central nervous system
ER: Estrogen receptor
ERE: Estrogen response element
GPER: G protein-coupled estrogen receptor
LH: Luteinizing hormone
MPOA: Medial preoptic area
OVX: Ovariectomy
PCOS: Polycystic ovary syndrome
PR: Progesterone receptor
PRE: Progesterone response element
PVH: Paraventricular nucleus of hypothalamus
VMH: Ventromedial nucleus of hypothalamus.
